# Incorporation of Obstacle Hardening into Local Approach to Cleavage Fracture to Predict Temperature Effects in the Ductile to Brittle Transition Regime

**DOI:** 10.3390/ma14051224

**Published:** 2021-03-05

**Authors:** Maria S. Yankova, Andrey P. Jivkov, Rajesh Patel

**Affiliations:** 1 Department of Mechanical, Aerospace and Civil Engineering, University of Manchester, Oxford Rd, Manchester M13 9PL, UK; andrey.jivkov@manchester.ac.uk; 2 National Nuclear Laboratory, Sperry Way, Stonehouse, GL10 3UT, UK; rajesh.patel@uknnl.com

**Keywords:** cleavage fracture, finite element analysis, local approach, Weibull stress

## Abstract

Ductile-to-brittle-transition refers to observable change in fracture mode with decreasing temperature—from slow ductile crack growth to rapid cleavage. It is exhibited by body-centred cubic metals and presents a challenge for integrity assessment of structural components made of such metals. Local approaches to cleavage fracture, based on Weibull stress as a cleavage crack-driving force, have been shown to predict fracture toughness at very low temperatures. However, they are ineffective in the transition regime without the recalibration of Weibull stress parameters, which requires further testing and thus diminishes their predictive capability. We propose new Weibull stress formulation with thinning function based on obstacle hardening model, which modifies the number of cleavage-initiating features with temperature. Our model is implemented as a post-processor of finite element analysis results. It is applied to analyses of standard compact tension specimens of typical reactor pressure vessel steel, for which deformation and fracture toughness properties in the transition regime are available. It is shown that the new Weibull stress is independent of temperature, and of Weibull shape parameter, within the experimental error. It accurately predicts the fracture toughness at any temperature in the transition regime without relying upon empirical fits for the first time.

## 1. Introduction

Metallic materials with body-centred cubic (bcc) lattices exhibit a unique behaviour with decreasing temperature—their fracture toughness decreases rapidly as the mode of fracture shifts from slow ductile crack growth, typically by void growth and coalescence, to fast brittle fracture, typically by transgranular cleavage. This behaviour is referred to as the ductile-to-brittle transition (DBT). A distinguishing feature of DBT is the scatter in the measured fracture toughness values, which arises from the random spatial distribution of microstructural features controlling the fracture processes. DBT presents a challenge for a number of industries, where structural components are made of bcc metals, with a very important example being ferritic steels. Stress-critical applications of ferritic steels include reactor pressure vessels (RPV) in light water reactors and pressurized equipment in hydrocarbon processing. These applications require reliable assessments of the fracture toughness in the DBT regime. Such assessments are challenging because the fracture mechanisms encompass multiple length scales—from atomic to component—and remain an active area of research.

The microstructure processes leading to ductile and cleavage failure modes are well known from experimental observations [1,2,3]. In ferritic steels, cleavage initiates at sharp microcracks formed in brittle carbides, sulphides, or other second phase particles. Alternatively, second phase particles could either decohere from the matrix or break and then blunt and form voids (prerequisites for ductile crack growth). Microstructural parameters of the particles such as their size, shape and orientation with respect to the applied load, have been shown to affect their tendency to break or decohere [1,2,3,4]. Additional factors such as irradiation and welds in the structural component need to be considered as they are known to modify the DBT behaviour. The main factor that determines the outcome in the competition between cleavage fracture and ductile tearing is the ability of the plastic deformation to relieve the applied stresses ahead of a crack. On a microstructural scale, plastic flow in bcc metals is controlled by the thermally activated movement of screw dislocations, which is dependent on a combination of an internal lattice resistance and an interaction between the dislocations and obstacles in the microstructure [5,6,7]. A recently published dislocation-obstacle model showed that the activation energy for plastic flow is mainly determined by the kink (which represents a step of atomic dimension in a dislocation line) formation energy [7]. Swinburne and Dudarev [7] were able to predict the ductile to brittle transition temperature in both unirradiated and irradiated ferritic-martensitic steels, where the irradiated temperature increased up to twice that of the unirradiated material in agreement with multiple experimental data. Their dislocation-obstacle interaction model is used as a key ingredient in our proposal.

From an engineering perspective, integrity assessments can be done by two different approaches. In the traditional global approach, a single mechanics parameter, such as the J integral, representing a crack-driving force, is evaluated against a material parameter—an experimentally obtained value representing the resistance to fracture, or the fracture toughness. This can be highly conservative, since the fracture toughness is typically obtained with a high-constraint (deep) crack, whereas the mechanics parameter could be obtained from analysis of a component with a low-constraint (shallow) crack. Apart from the inherent conservatism, the global approach cannot predict DBT behaviour—its use requires measurements of fracture toughness values at any temperature of interest and the decision which of the scattered values to evaluate against the crack driving force. 

Local approaches to cleavage fracture (LAF) were developed as alternatives to the global approach with the intention to capture the mechanistic understanding of the cleavage process described above. These are probabilistic models, which incorporate weakest link statistics of microcracks, controlled by local stresses, to model the observed scatter of fracture toughness values. The majority of local approaches to cleavage fracture are based on the Beremin model [8], which defines a microstructural crack-driving force, the so-called Weibull stress, and the probability of fracture as a Weibull distribution. The Weibull stress depends on two material-dependent parameters—a shape parameter m, linked to the shape of the probability density of the micro-crack sizes, and a scale parameter $\sigma_{u}$, linked to the elastic properties and surface energy of the material. Typically, the Weibull stress parameters are fitted to experimental data with shape parameters varying between 17 and 20 for different materials in the lower-shelf [9]. Considering that measured size distributions of second phase particles would provide significantly smaller Weibull shape parameters (around 4–5, see, e.g., [10]), such calibrations suggest large differences between the shapes of particle and micro-crack size distributions. 

While the Beremin model predicts well the cleavage fracture toughness in the lower-shelf of the DBT curve, the accurate prediction of cleavage fracture toughness in the DBT temperature regime has proven a challenge. Petti and Dodds [11] and Wasiluk et al. [12] proposed that the shape parameter should be independent of temperature, while the scale parameter should increase with temperature and should be calibrated to achieve agreement with the experimental data for the material. This is equivalent to the assumption that the shape of the micro-crack size distribution does not change with temperature, while the variation of cleavage fracture toughness is controlled by the variation of the material’s elastic properties and surface energy with temperature. That assumption is problematic because the changes of material’s elastic properties and surface energy are very small in the DBT temperature regime. Another option is to consider that the shape parameter changes with temperature, while the scale parameter is constant due to the argument in the previous sentence. For the case of small-scale yielding (SSY), the Hutchinson-Rice-Rosengren [13,14] fields have been used to calibrate the shape parameter in the DBT regime [15]. While the approach is reasonable, it still requires fracture toughness data at multiple temperatures, and assessments will be conservative, similarly to the global approach, due to the SSY conditions being used. The third option is to consider both Weibull stress parameters to be dependent on temperature. Wiesner and Goldthorpe [16] reported variation of *m* between 13 and 23 and of $\sigma_{u}$ between 1700 and 3700 MPa. As in the previous two cases, this option relies on extensive experimental data at a number of temperatures. A model for cleavage fracture toughness predictions in the DBT regime without relying on empirical fittings to a large number of toughness measurements is yet to be achieved. 

In the present study, we extend our previous approach [17] of incorporating a thinning function in the Weibull stress by presenting a theoretical formulation as opposed to an empirical one. The newly developed thinning function determines the number of cleavage initiators at a given temperature in the DBT regime. It is based on the kink-formation free energy from [7], and thus entirely theoretically based without any reliance on empirical fit to experimental data. We calibrated the model using a typical RPV steel with data from the Euro fracture dataset [18]. We were able to predict the characteristic fracture toughness at a few temperatures in the DBT regime based on experimental toughness data of a single temperature, which has not been done before. The paper is structured as follows: after a brief introduction to the Beremin model and some of the major previous modifications of the Weibull stress, we outline our new model and the required parameters, followed by a review of the finite element analysis in Section 2. Section 3 presents our results of applying the model and predicting the fracture toughness at three temperatures in the DBT region. Section 4 discusses the accuracy of the predictions and the importance of the findings, followed by a summary of the main conclusions in Section 5.

## 2. Methods

### 2.1. The Beremin Model

The Beremin model [8] was the first local approach to cleavage fracture, that is based on the weakest-link assumption, according to which the macroscopic cleavage event depends on the failure of a single cleavage initiating microcrack. This approximation is considered reasonable for the physical cleavage process, which consists of three main stages—firstly, microcracks initiate at randomly distributed second phase particles, assisted by the plastic deformation of the surrounding matrix; next, some microcracks propagate across the particle-matrix interface (alternatively, they can form voids, determined by the local mechanical conditions); and finally, cleavage occurs when a single microcrack propagates across the matrix without being arrested at a grain boundary [19,20]. From a statistical point of view, the cleavage process is represented as an inhomogeneous spatial Poisson point process [21] with points corresponding to the cleavage initiators (CI). There are three conditions to be met for a Poisson process including: failures of non-overlapping volumes are independent of each other; the probability of failure of a unit volume is proportional to its volume and the probability of more than one failure in the unit volume is zero. If $\mu_{c}$ is the probability density of sharp microcracks eligible for cleavage, the probability of failure $\delta P_{f}$ of a unit volume δV and that of the whole solid $P_{f}$ are:

(1) $$\delta P_{f}=\mu_{c}\delta V=\left(\frac{1}{V_{0}}\int_{a_{c}}^{\infty }f_{c}\left(a\right)da\right)\delta V\;\mathrm{and}$$

(2) $$P_{f}=1-\exp\left(-\int_{V}\mu_{c}dV\right),\;$$

where $f_{c}\left(a\right)$ is the microcrack size distribution, $a_{c}$ is the critical size of a penny-shaped crack of size a according to Griffith’s criterion, and $V_{0}$ is a reference volume. The critical microcrack size can therefore be expressed as:

(3) $$a_{c}=\frac{\pi E\gamma }{2\left(1-\nu^{2}\right)\sigma_{1}^{2}},$$

where E and ν are the elastic modulus and Poisson’s ratio of the steel, respectively, $\sigma_{1}$ is the maximum principal stress, and γ is a measure of fracture energy. 

In the original Beremin model, plastic deformation is assumed to be a prerequisite for cleavage initiation, and the microcrack size distribution is assumed to follow a power law with scale and shape parameters, β and α, as follows:

(4) $$f_{c}\left(a\right)=\theta {\left(\frac{\beta }{a}\right)}^{\alpha },$$

where θ, the fraction of particles converted into eligible micro-cracks, is equal to the Heaviside step function, $\theta =H\left(\varepsilon_{p}\right)$, or in other words all cleavage initiators nucleate at the onset of plasticity. In the terminology of the statistical Poisson process, θ is a thinning function, which creates a new Poisson process by only including some of the points of the original Poisson process. Integrating the microcrack size distribution and rearranging leads to the expression for the total failure probability in terms of the Weibull stress $\sigma_{w}$:

(5) $$P_{f}\left(\sigma_{w}\right)=1\;-\exp{\left(\frac{\sigma_{w}}{\sigma_{u}}\right)}^{m},\;\mathrm{and}$$

(6) $$\sigma_{w}={\left(\frac{1}{V_{0}}\int_{V}\theta \sigma_{1}^{m}dV\right)}^{1/m},\;$$

where the Weibull shape and scale parameters are: m=2α−2, and $\sigma_{u}={\left(\frac{\pi E\gamma }{2\left(1-\nu^{2}\right)\beta }\right)}^{1/2}$, respectively. We note that the shape parameter m is linked to the shape parameter of the microcrack size distribution, where it is assumed that the distribution follows the same shape as that of the particle size distribution, whereas the scale parameter $\sigma_{u}$ incorporates the elastic properties, the surface energy, and the scale of the size distribution.

### 2.2. Proposed Model

In bcc metals, the plastic flow is controlled by the thermally activated motion of screw dislocations and their interaction with obstacles. Dislocations move through the lattice via kinks, minimizing the Peierls potential of the dislocation. Swinburne and Dudarev [7] developed a model of obstacle hardening of bcc materials that accounts for the kink mechanism, which is temperature and shear stress dependent, in the dislocation-obstacle interaction. Their model demonstrated that the kink activation energy halves when a critical length becomes smaller than the average dislocation segment.

Equations (7) and (8) show the critical length $L^{*}$ and the kink formation free energy $F_{k}$, analogous to [7], where we substitute the maximum shear stress using Tresca criterion as an approximation to the Peierls stress, b is the dislocation Burger’s vector, T is the temperature, k is the Boltzmann’s constant, $\sigma_{p}$ is the critical Peierls stress, $T_{\mathrm{ath}}$ is the athermal temperature and we set the free energy to zero in elements with negative energy due to numerical reasons:

(7) $$L^{*}\left(\tau_{\max},T\right)=b\;\exp\left[F_{k}\left(\tau_{\max},\;T\right)/kT\right],$$

(8) $$F_{k}\left(\tau_{\max},\;T\right)=\;\{\begin{array}{c} \;\;\;\;\;\;\;\;\;\;\;\;\;\;\;\;\;\;\;\;\;\;\;\;\;\;0.0,\;\;\;\;\;\;\;\;\;\;\;\;\;\;\;\tau_{\max}>\sigma_{p}{\left(1\;-T/T_{\mathrm{ath}}\right)}^{2}\\ U_{k}\left(1-\frac{T}{T_{\mathrm{ath}}}-\frac{\tau_{\max}/\sigma_{p}}{1\;-T/T_{\mathrm{ath}}}\right),\;\tau_{\max}<\sigma_{p}{\left(1\;-T/T_{\mathrm{ath}}\right)}^{2} \end{array}.\;$$

Setting the average dislocation segment to the average distance between carbides ⟨d⟩, we calculate a normalized free energy as:

(9) $$f_{k}\left(\tau_{\max},T\right)=\{\begin{array}{c} 2F_{k}\left(\tau_{\max},T\right)/kT,\;\;\;L^{*}\geq \;\langle d\rangle \\ F_{k}\left(\tau_{\max},T\right)/kT,\;\;\;\;\;\;\;L^{*}\leq \langle d\rangle \end{array}.$$

Next, we obtain a thinning function θ for the Poisson process, dependent on the normalized free energy as:

(10) $$\theta \left(\tau_{\max},T\right)=\frac{f_{k}\left(\tau_{\max},T\right)}{f_{k}\left(0\;\mathrm{MPa},-200\;{}^{\circ}C\right)}.$$

**Figure 1:**
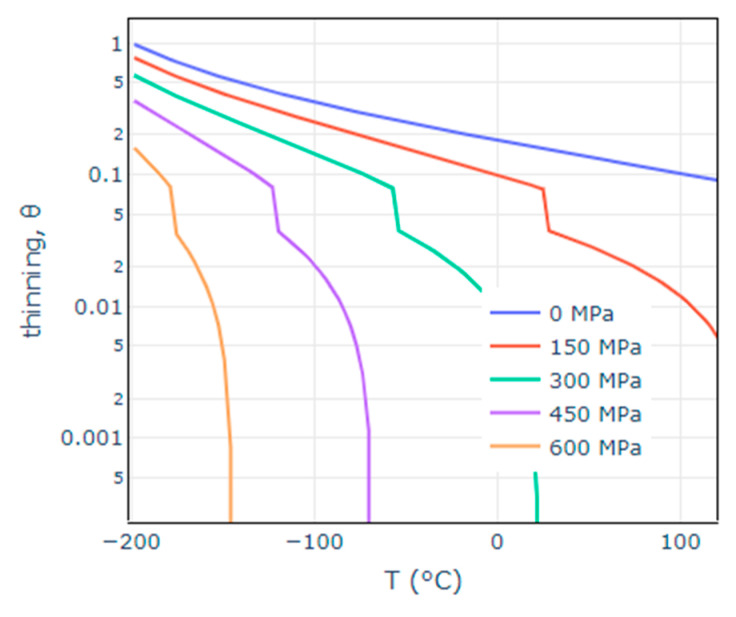
Poisson thinning vs. temperature for a range of shear stresses.

The as-constructed thinning function’s behaviour is shown in Figure 1 as a function of the shear stress and temperature. At very low temperatures and stresses, the thinning tends to 1, which corresponds to all particles being converted to cleavage initiators, whereas increasing the temperature and the maximum shear stress reduces the value of the thinning as physically expected by the reduction in particles that form sharp micro-cracks.

**Figure 2:**
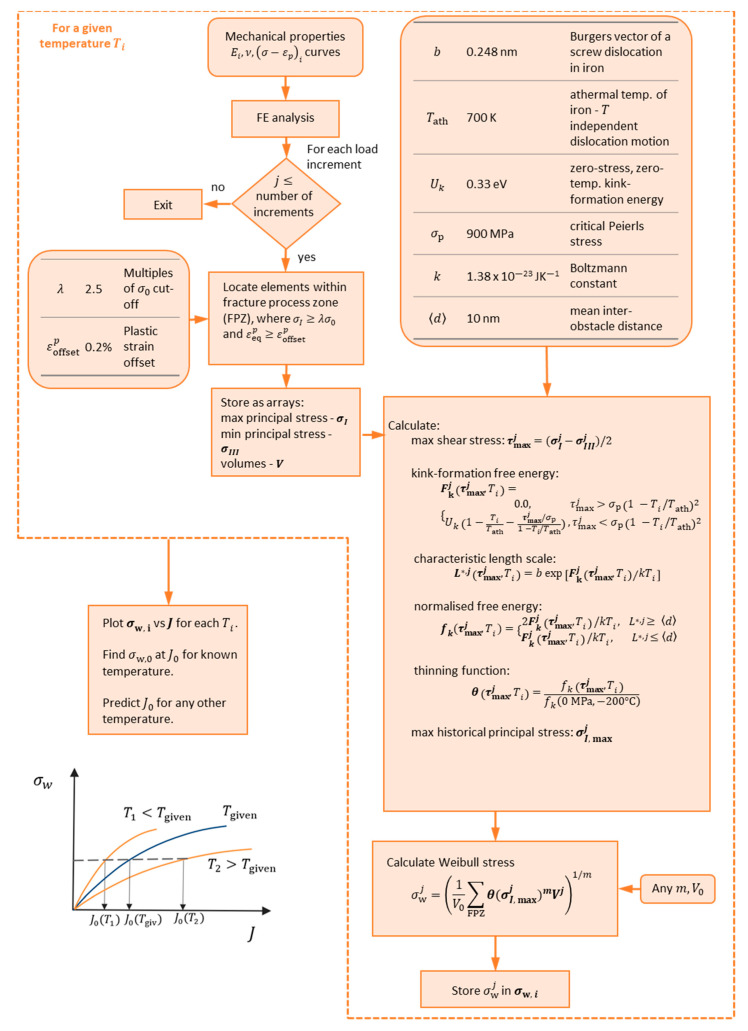
Flow chart of the developed model.

Figure 2 presents a flow chart of the developed model—for a given temperature $T_{\mathrm{given}}$, at which at least 8 to 10 fracture toughness measurements are available, we perform a finite element (FE) analysis and for each load increment, we find the elements within fracture process zone (FPZ), based on two conditions for the maximum principal stress and the equivalent plastic strain, typically applied in the local approaches to fracture, as follows: $\sigma_{I}\geq \lambda \sigma_{0}$ and $\varepsilon_{\mathrm{eq}}^{p}\geq \varepsilon_{\mathrm{offset}}^{p}$, where λ is a scalar equal to between 1 and 2.5, and $\varepsilon_{\mathrm{offset}}^{p}$ is equal to 0.2%. The maximum $\sigma_{I}$ and minimum $\sigma_{I\;II}$ principal stresses and the volumes V for all elements in the FPZ are stored as arrays. Then, the local approach is applied as a post-processor to the FE data—we loop over all load increments and for each increment calculate the maximum shear stress based on Tresca criterion, the kink-formation free energy and characteristic length scale [7], the normalized free energy (Equation (9)) and the thinning function (Equation (10)), the maximum principal stress within the load history up to the current increment. Next, we compute the Weibull stress using *any* shape parameter m and unit volume $V_{0}$ (here, set to the volume of a spherical grain with a 10 μm radius, which corresponds to the approximate grain size in RPV steels [22]). The procedure is repeated for any temperature of interest $T_{i}$, at which we would like to predict the fracture toughness. Finally, we plot the Weibull stress curves as a function of J as shown in the schematic in Figure 2 and compute the predicted J for a given percentile, for instance the characteristic $J_{0}$ at 63.2%, at each temperature $T_{i}$ corresponding to the equivalent Weibull stress for the given temperature. There is no need to calibrate a scale parameter $\sigma_{u}$, since the probability curve at $T_{\mathrm{given}}$ can be used to find the corresponding Weibull stress at each required J percentile, and therefore, find this fracture toughness percentile for each temperature $T_{i}$.

### 2.3. Finite Element Analysis

The model was developed using experimental fracture toughness and mechanical properties for the reactor pressure vessel steel 22NiMoCr37, which are available in the literature within the lower shelf and in DBT. Fracture toughness data [18] for a standard 1T compact tension specimen with thickness B = 25 mm, width W = 50 mm and crack length a to width W ratio of 0.5 at three temperatures, −91 °C, −60 °C and −40 °C, were used. The toughness data were ranked in ascending order with a rank probability $F\left(J_{c}^{i}\right)=\left(i-0.3\right)/\left(N+0.4\right)$, where *i* = 1, …, *N* and N is the total number of data points. The temperature dependence of the Young’s modulus E, the yield stress $\sigma_{Y}$ and the ultimate tensile stress $\sigma_{\mathrm{UTS}}$ were approximated by [23]:

(11) E=−90T+206,000,

(12) $$\sigma_{Y}=421.2+63.9\exp\left(-T/91\right)\;\mathrm{and}$$

(13) $$\sigma_{\mathrm{UTS}}=564.1+70.2\exp\left(-T/108\right).$$

**Figure 3:**
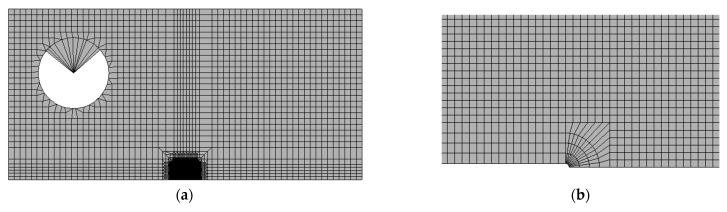
(**a**) 2D half 1T compact tension specimen model; (**b**) Uniform mesh design with a finite radius crack tip.

Abaqus 2017 [24] was used to model two-dimensional models of a half standard 1T compact tension specimen, 1T-C(T) with the above material properties in the large strain finite element analyses. A mesh with finite crack tip radius was designed, where the corresponding radius for each temperature was chosen approximately five times smaller than the crack tip opening displacement at the characteristic fracture toughness $J_{0}$. The radii equal to 10, 15 and 20 μm for −91 °C, −60 °C and −40 °C, respectively, were verified against boundary layer models in a previous study for each of the corresponding temperatures [25]. Figure 3a shows the half-C(T) model with a 10-μm radius. The local approaches have been found to be more sensitive to the mesh design compared to the global approaches. Thus, we designed a mesh with a uniform element size within the area of interest, as shown in Figure 3b. There is a small fan section of the mesh near the crack tip with a side of no more than five times the initial radius, which is excluded from the Weibull stress calculation based on the yield stress cut-off condition specified above. This uniform mesh design was shown to lead to a more consistent Weibull stress calculations across temperatures compared to a traditional fan mesh due to the change of the stress and strain fields ahead of the crack with increasing temperatures. The experimental high-strength maraging steel loading pin [26] was replaced by a purely elastic wedge, which was found as a good approximation and provided shorter computational times. The material was modelled as elastic-plastic with flow properties computed using Equations (11)–(13) and specified in a tabular form in the FE software.

## 3. Results

**Figure 4:**
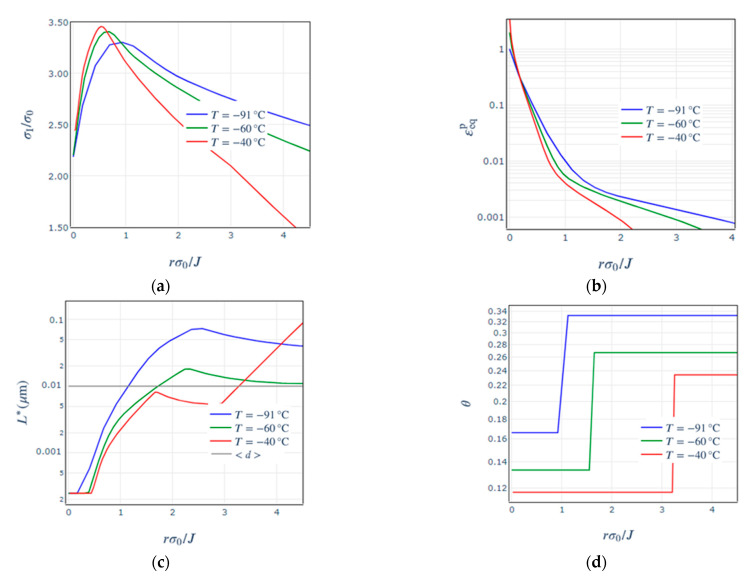
(**a**) Normalised maximum principal stress, (**b**) equivalent plastic strain, (**c**) critical length and mean interparticle distance; and (**d**) thinning as a function of the normalized distance from the crack tip for the 1T C(T) models at all temperatures.

Figure 4a,b show the normalised maximum principal stresses and the plastic strain fields ahead of the crack tip, where the distance from the crack tip has been normalized as well. These mechanical parameters behave as expected with the increase of temperature. The critical length $L^{*}$ as calculated per Equation (7) decreases with increasing temperature, as seen in Figure 4c. It represents the effect of easier dislocation motion through the lattice at higher temperatures due to a reduced lattice resistance, and accordingly an increase in the plastic flow. The average dislocation segment ⟨d⟩ was assumed to be equal to 10 nm and is indicated with a grey line. The thinning ahead of the crack is also plotted in Figure 4d, where we note the step of doubling the value at the critical length $L^{*}$ crossing ⟨d⟩.

**Figure 5:**
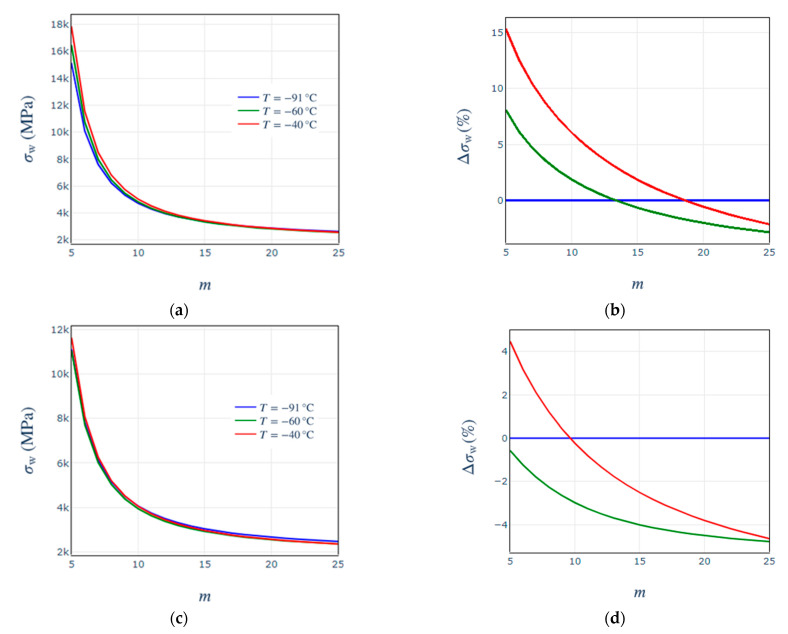
(**a**) Weibull stress and (**b**) Weibull stress difference in percentage with respect to the Weibull stress at −91 °C, using the original Beremin model as a function of the shape parameter m; (**c**) Weibull stress and (**d**) Weibull stress difference in percentage with respect to the Weibull stress at −91 °C, using the developed model as a function of the shape parameter m.

Figure 5 presents the Weibull stress calculated for each of the three temperatures considered as a function of the shape parameter m using the original Beremin model in (a) and using the developed model in (c). Furthermore, the Weibull stress differences between the two higher temperatures and the lowest temperature are obtained, as shown in Figure 5b,d, for the original and the developed models, respectively. Qualitatively, we observe the Weibull stress curves to diverge, specifically at low m values, when the original Beremin model is used. On the contrary, the developed model brings the curves to coincide. The importance of this coincidence is that the proposed model works with any shape parameter within reasonable limits, or inversely, that the shape parameter to be used in cleavage fracture toughness predictions is independent of temperature. Quantitatively, we observe a reduction in the difference between the Weibull stress curves from up to about 10% in the case of the original Beremin model, to up to about 4.5% in the case of the developed model. This is within the uncertainty limits for the deformation properties of the material measured at different temperatures and used for the analyses in the work.

**Figure 6:**
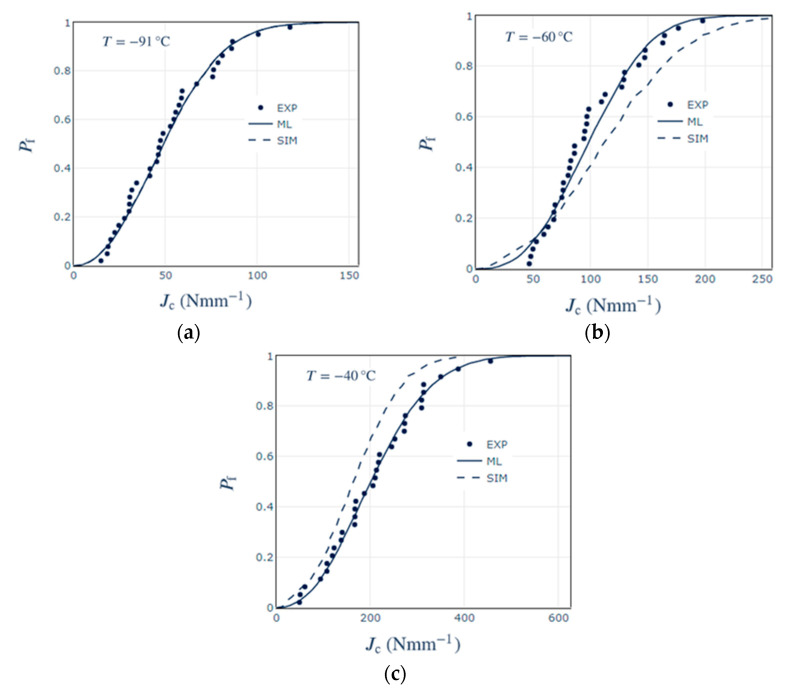
Cumulative probability function of experimentally measured $J_{c}$ using the Maximum Likelihood (ML) method and Weibull fit as predicted by the proposed method for (**a**) T = −91 °C, (**b**) T = −60 °C and (**c**) T = −40 °C, where the toughness data at T = −91 °C is used for calibration of the model at *m* = 7.

Next, we consider the characteristic fracture toughness predictions, as listed in Table 1. There is a good agreement between the measured and the predicted values using simulations. Figure 6 presents the cumulative probability distribution fits using the Maximum Likelihood (ML) method to the experimentally measured $J_{c}$ as well as the simulated fits based on a calibration of the developed model to the toughness data at T = −91 °C and a Weibull shape parameter *m* = 7. At the fitted temperature of −91 °C, there is a complete coincidence with the experimentally fitted curve. At temperatures of −60° and −40 °C, there is better agreement at the lower toughness values, with some under- and overestimation at the higher toughness values, respectively. This is due to the larger number of experimental data points at the lower toughness values at −91 °C, and so there is a better agreement with the predicted values. 

## 4. Discussion

We showed previously [17,24] that the mechanical fields alone are not enough to capture the large exponential difference between the Weibull stress curves, which in general do not cross if no correction is applied. This means the use of a single shape parameter m would be impossible to predict the toughness across temperatures. Additionally, under the assumption of cleavage fracture as a Poisson point process, it was found that for the same percentile *J*_p_, the number of cleavage initiators are equal across different temperatures, but they are spread across a different plastic zone volume, i.e., the volume density of CI changes. The proposed thinning function mathematically captures this effect—it represents the decrease of microcracks that convert to CI with the increase of temperature. As shown in Figure 1, the thinning is close to one at low temperatures and shear stresses, corresponding to the case of all microcracks to convert into cleavage initiators. As the temperature and the shear stress (corresponding to the increase of plasticity due to loss of constraint) increase the thinning function reduces, and so a larger fraction of the microcracks will convert into voids rather than cleavage initiators. The biggest advantage of the current form of the thinning function and the developed model is the theoretical basis and so lack of empirical fittings required. The model includes microstructural parameters of the crystal structure of iron and the Boltzmann constant, as shown in Figure 2. All constant parameters have been computed for the screw dislocation in iron in the literature using atomistic simulations [4]. The only parameter calibrated as part of the model is the mean inter-obstacle distance—the value was chosen to describe an assumed mean inter-obstacle distance for the microstructure of a typical RPV steel and it did not have significant effects on the results. The model also includes two engineering parameters, which have values typically chosen in the local approaches to cleavage models as listed in Figure 2, namely the multiples of $\sigma_{0}$ cut-off and the plastic strain offset.

The major benefit of the developed local approach to cleavage fracture is the ability to predict the probability of cleavage at any temperature in the ductile-to-brittle regime by calibration through fracture toughness experiments at a single temperature. Previous approaches [9,15] require fracture toughness experiments at multiple temperatures, which is costly and time consuming. The developed model is of great industrial relevance, as it reduces the cost and time of performing these assessments.

Furthermore, the developed local approach to cleavage has the potential to predict the changes of fracture toughness due to irradiation. It is known that the macroscopic effects of irradiation are increased yield stress and reduced hardening and ductility. In fact, the kink formation energy model [7] used here has been developed to predict precisely these changes with irradiation. The application of the proposed model promises to capture the change of fracture toughness solely due to these changes of deformation properties, because at a given temperature the changes will result in reduced shear stress and from there in increased density of eligible micro-cracks in the plastic zone, leading eventually to higher probability of cleavage compared to unirradiated material at the same temperature. This needs to be explored in future work, although the validation would be even more challenging due to the very limited fracture toughness data of irradiated materials.

## 5. Conclusions

The main findings of this study are summarised below:

- An exponential correction to the density of micro-cracks eligible for cleavage, which is dependent on a material property, that changes with temperature, was proposed. It represents a thinning function of the Poisson process, which creates a new Poisson process with a subset of the points of the original Poisson process.
- The developed procedure for probability of cleavage estimation would only require experimental fracture toughness tests at a given temperature, and deformation properties at any other temperature of interest.
- Very good agreement was shown between the predicted and experimentally measured characteristic fracture toughness as well as the predicted and experimentally fitted probability distributions.
- The value of the work stems from the possibility of significant reduction in the necessary experimental fracture toughness testing.
- The validation of the model requires four to six deformation tests, and at least 30 fracture toughness tests at three constraint conditions with a ratio, a/w of 0.5, 0.2 and 0.1.
- These would allow the prediction of cleavage toughness in the ductile to brittle transition regime using a decoupled model, which makes it advantageous for engineering purposes.

## Figures and Tables

**Table 1:** Characteristic fracture toughness *J*_0_ calculated based on a Weibull distribution fit to the experimental data using the maximum likelihood method and predicted using the developed method based on the fracture toughness experimental data at T = −91 °C, −60 °C and −40 °C, and a shape parameter *m* = 10.

*T* (°C)	$J_{0}^{\mathrm{EXP}}$ (Nmm^−1^)	$J_{0}^{\mathrm{SIM},\;T=-91\;{}^{\circ}C}$ (Nmm^−1^)	$J_{0}^{\mathrm{SIM},\;T=-60\;{}^{\circ}C}$ (Nmm^−1^)	$J_{0}^{\mathrm{SIM},\;T=-40\;{}^{\circ}C}$ (Nmm^−1^)
−91	57.6	-	41.0	57.5
−60	112.3	153.8	-	153.8
−40	234.9	237.6	177.2	-

## Data Availability

Data supporting the findings of this study are available from the corresponding author upon request.
